# Baicalein and Baicalin Promote Melanoma Apoptosis and Senescence *via* Metabolic Inhibition

**DOI:** 10.3389/fcell.2020.00836

**Published:** 2020-08-25

**Authors:** Lan Huang, Bo Peng, Yash Nayak, Cindy Wang, Fusheng Si, Xia Liu, Jie Dou, Huaxi Xu, Guangyong Peng

**Affiliations:** ^1^Department of Immunology, School of Medicine, Jiangsu University, Zhenjiang, China; ^2^Division of Infectious Diseases, Allergy and Immunology, Department of Internal Medicine, School of Medicine, Saint Louis University, Saint Louis, MO, United States; ^3^State Key Laboratory of Natural Medicines, School of Life Science and Technology, China Pharmaceutical University, Nanjing, China

**Keywords:** baicalein, baicalin, melanoma, N-RAS, B-RAF, apoptosis, senescence, glucose metabolism

## Abstract

Malignant melanoma is one of the most common and dangerous skin cancers with a high rate of death every year. Furthermore, N-RAS and B-RAF mutations in melanoma cells increase the difficulties for clinical treatment in patients. Therefore, development of effective and universal drugs against melanoma is urgently needed. Here we demonstrate that baicalein and baicalin, the active components of the Chinese traditional medicinal plant *Scutellaria baicalensis* Georgi, can significantly inhibit melanoma cell growth and proliferation, suppress tumor cell colony formation and migration, as well as induce apoptosis and senescence in melanoma cells. The anti-tumor effects mediated by baicalein and baicalin are independent of N-RAS and B-RAF mutation statuses in melanoma cells. Mechanistically, we identify that the suppression of baicalein and baicalin on melanoma cells is due to inhibition of tumor cell glucose uptake and metabolism by affecting the mTOR-HIF-1α signaling pathway. In addition, we demonstrated that baicalein and baicalin can suppress tumorigenesis and tumor growth *in vivo* in the melanoma model. These studies clearly indicate that baicalein and baicalin can control tumor growth and development metabolically and have great potential as novel and universal drugs for melanoma therapy.

## Introduction

Melanoma, a type of skin cancer, is one of the deadly cancers in the world. In the United States, according to the American Cancer Society, there were 91,270 new cases of melanoma, and 9,320 cases were expected to die in 2018 (American Cancer Society, 2018). Great progress has made in diagnosis and treatment with melanoma, but the overall survival of advanced melanoma is still very low. In addition, melanoma with N-RAS and B-RAF mutations has been well-recognized as a challenge that brings out more difficulties for the treatment (Brose et al., 2002; Omholt et al., 2003; Thumar et al., 2014). Furthermore, the status of those mutations is directly associated with the worse prognosis of the cancer patients (Thomas et al., 2015). Although certain activated signaling pathways induced by the mutations, such as RAS-RAF-MAPK have been identified as the promising targets for drug development, the clinical therapeutic effects of target inhibitors are still varied (Flaherty et al., 2010; Nazarian et al., 2010; Kaplan et al., 2011). Importantly, treatments with some target inhibitors may induce drug resistance and/or promote tumor growth and progression in cancer patients as well as pre-clinical tumor models (Flaherty et al., 2010; Hatzivassiliou et al., 2010; Nazarian et al., 2010; Poulikakos et al., 2010; Kaplan et al., 2011; Kwong et al., 2012). Therefore, development of the unique inhibitors to target specific mutations or universal drugs against melanoma is urgently needed.

Recent studies suggest that many natural products have been developed into promising drugs and applied into clinical trials for various disease treatments including cancers (Cragg et al., 2009; Cragg and Newman, 2013; Stover et al., 2014). Baicalin and its aglycon baicalein are the major flavonoids derived from the edible medicinal plants *Scutellaria baicalensis* Georgi (Xiao et al., 2014). Baicalein and baicalin have been widely used for inflammation and infectious disease treatments (Johnson, 2011; Ding et al., 2014; Moghaddam et al., 2014; de Oliveira et al., 2015; Ji et al., 2015). Furthermore, both baicalein and baicalin are also potent anti-tumor drugs, which have been shown strong anti-tumor effects in various cancers, including in breast cancer, prostate cancer, pancreatic cancer, esophageal squamous cell carcinoma and burkitt lymphoma (Takahashi et al., 2011; Huang et al., 2012; Yu et al., 2013; Zhang et al., 2013; Aryal et al., 2014; Chung et al., 2015; Dou et al., 2018). Both compounds can inhibit the proliferation, migration, adhesion and invasive properties of tumor cells, and induce tumor cell cycle arrest (Chao et al., 2007; Chiu et al., 2011; Takahashi et al., 2011; Aryal et al., 2014; Wang et al., 2015; Gong et al., 2017). We have recently demonstrated that baicalein and baicalin could inhibit human colon cancer cell growth and proliferation *in vitro* and *in vivo* (Dou et al., 2018; Wang et al., 2018). The suppressive effects are due to the induction of colon cancer cell apoptosis and senescence (Dou et al., 2018; Wang et al., 2018). However, whether baicalein and baicalin have anti-tumor effects against melanoma, especially melanoma with mutations is unknown. Furthermore, the molecular mechanism by which the two compounds inhibit cancer is still unclear. A precise understanding of biological functions and mechanisms of these two natural compounds on different types of cancers will provide novel targets for the clinical therapy against cancers including melanoma.

In this study, we explored the anti-tumor effects and related mechanism of baicalein and baicalin in melanoma. We demonstrated that baicalein and baicalin can significantly inhibit both human and mouse melanoma cell growth and proliferation, suppress tumor cell colony formation and migration, as well as induce apoptosis and senescence in melanoma cells. The anti-tumor effects mediated by baicalein and baicalin are independent of N-RAS and B-RAF mutation statuses in melanoma cells. Furthermore, we identified that the suppressive effects mediated by baicalein and baicalin on tumor cells are mechanistically due to the inhibition of tumor cell glucose metabolism, which are molecularly controlled by mTORC1-HIF-1α signaling pathway in melanoma cells. In addition, we demonstrated that baicalein and baicalin can suppress tumorigenesis and tumor growth *in vivo* in the melanoma model. These studies clearly indicate that baicalein and baicalin could be potential novel and universal drugs for melanoma therapy.

## Results

### Baicalein and Baicalin Inhibit Melanoma Cell Growth and Proliferation

**FIGURE 1:**
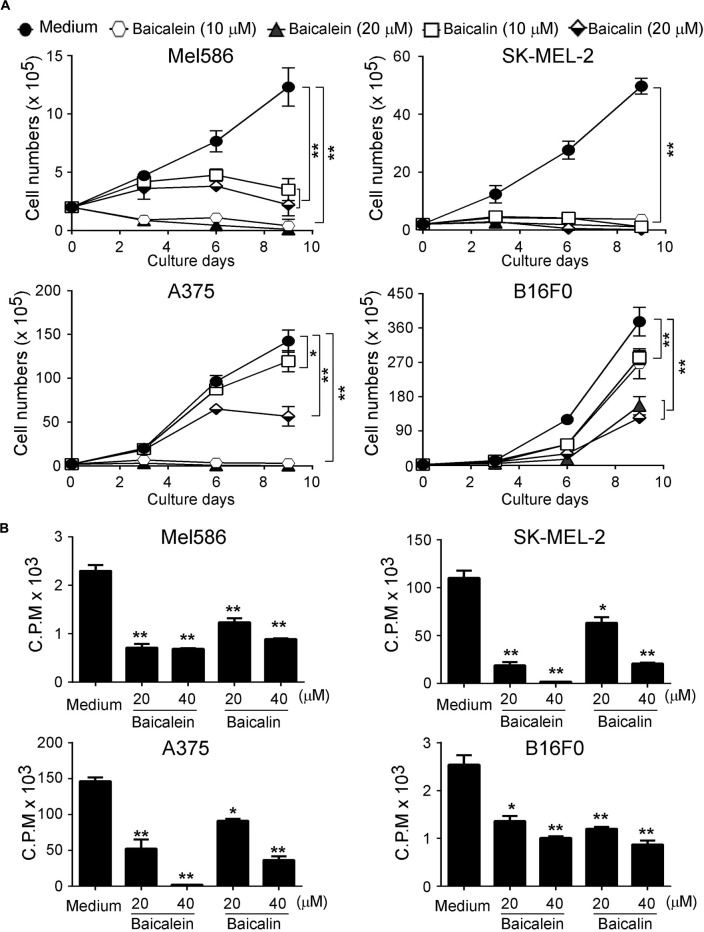
Baicaleinand baicalin inhibit both melanoma cell growth and proliferation. Three human melanoma cell lines (Mel586, SK-MEL-2, and A375) and one mouse melanoma cell line (B16F0) were cultured at a started number of 2 × 10^5^/well in 6-well plates, or 5 × 10^3^/well in 96-well plates, and treated with the indicated concentrations of baicalein or baicalin. The cell growth was evaluated at different time points using the cell number counting **(A)**, and cell proliferation was determined using [^3^H]-thymidine assays **(B)**. Data shown in Panels **(A,B)** are mean ± SD from three independent experiments with similar results. *p < 0.05 and **p < 0.01 compared with the medium control group.

Our previous studies have demonstrated that baicalein and baicalin can suppress colon cancer cell proliferation and growth (Dou et al., 2018; Wang et al., 2018). We further determined whether baicalein and baicalin can inhibit tumor growth of melanoma cells. Three human melanoma cell lines Mel586, SK-MEL-2 (wild type B-RAF and mutant N-RAS), A375 (B-RAF V600E and wild type N-RAS), as well as mouse B16F0 melanoma cell line were cultured in the presence of different concentrations of baicalein and baicalin. Tumor cell growth and proliferation were further determined using cell growth curve and [^3^H]-thymidine incorporation assays. We found that baicalein and baicalin strongly suppressed tumor growth and proliferation of both human and mouse melanoma cells (Mel586, SK-MEL-2, A375 and B16F0) regardless of the mutation statuses of B-RAF and N-RAS (Figures 1A,B). Furthermore, the suppressive effects mediated by the two compounds were in a dose-dependent manner. In addition, baicalein has stronger inhibitory activity on melanoma cell growth and proliferation than that of baicalin. High concentration of baicalein (40 μM) almost completely inhibited human melanoma growth (Figure 1B). These results clearly suggest that both baicalein and baicalin strongly suppress melanoma cell proliferation and growth.

### Baicalein and Baicalin Inhibit Melanoma Cell Colony Formation, Migration and Adhesion

**FIGURE 2:**
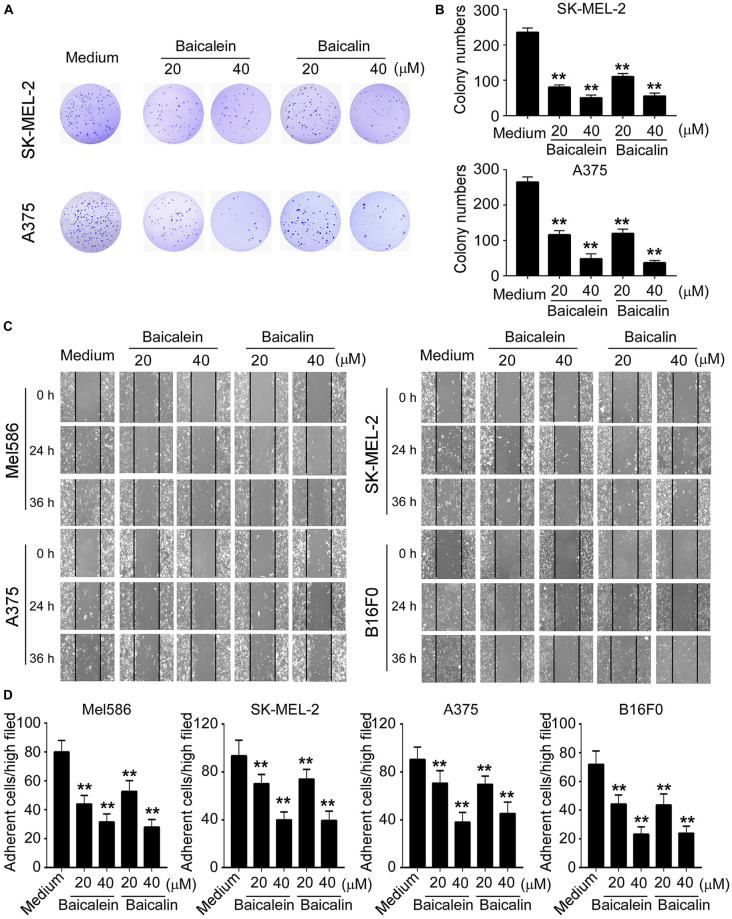
Baicalein and baicalin inhibit melanoma cell colony formation, migration and adhesion. **(A,B)** Baicalein and baicalin treatments dramatically decreased the numbers and sizes of tumor colonies in SK-MEL-2 and A375 cells. 200–500 per well of melanoma cells pre-treated with the indicated concentrations of baicalein or baicalin, were seeded in 6-well plates for culture, and cell colonies counted after 10–14 days of culture. Results shown in the histogram **(B)** are summaries of mean ± SD from three independent experiments. **p < 0.01 compared with the medium control group. **(C)** Different concentrations of baicalein and baicalin treatments in both human and mouse melanoma cells significantly inhibited tumor cell migration compared with the medium control group at 24 and 36 h time points in the wound closure assays. Data shown are representatives from three independent experiments with similar results. **(D)** Baicalein and baicalin treatments suppressed the adhesion of melanoma cells. Both human and mouse melanoma cells pretreated with the indicated concentrations of baicalein and baicalin were cultured in the fibronectin-coated plates for 45 min. Adherent cells were counted and averaged in 10 fields at high (×400) magnification with a microscope. Results shown are summaries of mean ± SD from three independent experiments with similar results **p < 0.01 compared with the medium control group.

We then investigated whether baicalein and baicalin have inhibitory effects on the other key biological behaviors of melanoma cells, including colony formation, migration and adhesion capacities. We observed that the numbers and sizes of tumor cell colonies were significantly decreased in SK-MEL-2 and A375 tumor cells after treatment with baicalein or baicalin (Figures 2A,B). Furthermore, two different concentrations of baicalein and baicalin markedly suppressed both human and mouse melanoma cell migration at different time points using a wound healing assay (Figure 2C). In addition, baicalein and baicalin inhibited the adhesion ability of both human and mouse melanoma cell lines (Figure 2D). Consistent with the effect on tumor growth and proliferation, the suppression of melanoma cell colony formation, migration and adhesion is in a dose-dependent manner and regardless of the mutation statuses of B-RAF and N-RAS genes. These results collectively indicate baicalein and baicalin can strongly inhibit tumor cell growth and biological behaviors in melanoma.

### Baicalein and Baicalin Induce Apoptosis and Senescence in Melanoma Cells

**FIGURE 3:**
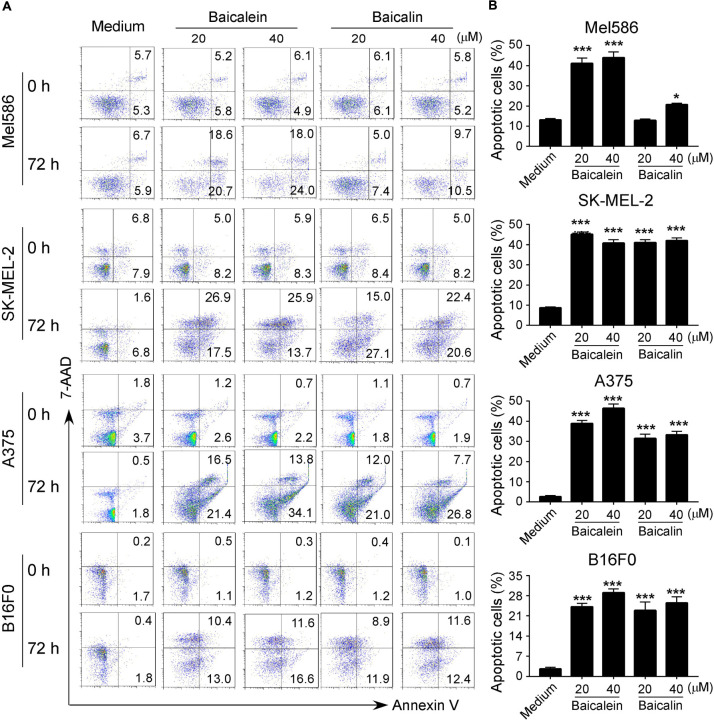
Baicalein and baicalin treatments promote melanoma cell apoptosis. **(A,B)** Significantly increased apoptotic cell populations were induced in both human and mouse melanoma cells after treatments with baicalein and baicalin. Tumor cells were cultured in the presence of indicated concentrations of baicalein and baicalin for 72 h. Apoptosis in treated tumor cells was analyzed after staining with PE-labeled Annexin V and 7-AAD **(A)**. Results shown in the histogram **(B)** are summaries of mean ± SD from three independent experiments. *p < 0.05 and ***p < 0.001, compared with the medium control group.

Our previously studies have shown that baicalein and baicalin can induce colon cancer cell apoptosis and senescence (Dou et al., 2018; Wang et al., 2018). Furthermore, studies from other groups have also shown that baicalein and baicalin induce cancer cell apoptosis in pancreatic cancer, esophageal squamous cell carcinoma and burkitt lymphoma (Takahashi et al., 2011; Huang et al., 2012; Zhang et al., 2013). Therefore, we measured apoptosis and cell death in melanoma cell lines treated by both baicalein and baicalin. We found that culture with medium, melanoma cells contained around 2–10% apoptotic cells (around 10% in Mel586 and SK-MEL-2, and below 5% in A375 and B16F0 tumor cells). Furthermore, consistent with the previous reports in other tumor cells, treatment with baicalein significantly induced tumor cell apoptosis in both human and mouse melanoma cells at 36 and 72 h (Figures 3A,B and Supplementary Figure S1) (Takahashi et al., 2011; Huang et al., 2012; Zhang et al., 2013; Dou et al., 2018; Wang et al., 2018). We have previously shown that baicalin treatment induced increased senescence rather than apoptosis or cell death in colon cancer cell lines (Dou et al., 2018; Wang et al., 2018). However, our current studies clearly showed that treatment with baicalin also dramatically induced tumor cell apoptosis in both human and mouse melanoma cells, but its effect is less potent than that of baicalein (Figure 3 and Supplementary Figure S1). Interestingly, treatments with baicalein and baicalin also induced some necrotic cell populations in SK-MEL-2 and B16F0 tumor cells (Figure 3A).

**FIGURE 4:**
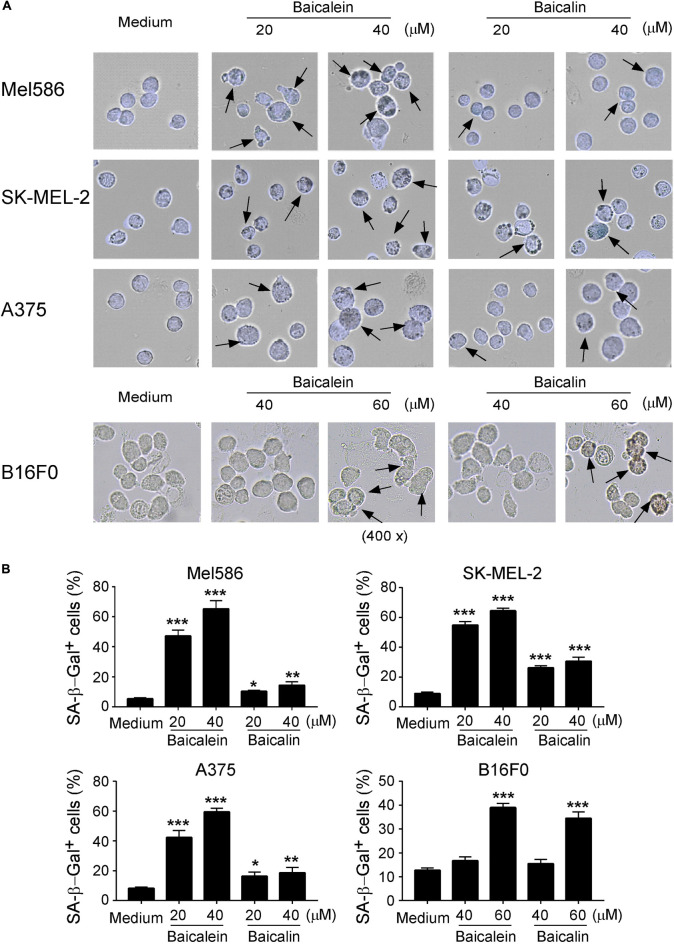
Baicalein and baicalin treatments induce melanoma cell senescence. **(A,B)** Increased senescent cell populations were induced in human and mouse melanoma cells after treatments with baicalein and baicalin. Tumor cells were cultured in the presence of indicated concentrations of baicalein and baicalin for 72 h. Senescent cells were analyzed using the SA-β-Gal activity assay and the SA-β-Gal positive cells were identified with dark blue granules as indicated by the arrows **(A)**. Data in panel **(B)** are mean ± SD from three independent experiments with similar results. *p < 0.05, **p < 0.01, and ***p < 0.001, compared with the medium control group.

We then determined whether baicalein and baicalin can induce senescence in melanoma cells, which is another mechanism involved in the suppressed cell growth and proliferation mediated by baicalin in colon cancer cells (Dou et al., 2018; Wang et al., 2018). The most widely used biomarker for senescent cells is the senescence-associated β-galactosidase (SA-β-Gal) (Ye et al., 2012; Liu et al., 2018). We observed that culture with baicalein and baicalin in human Mel586, SK-MEL-2, and A375 melanoma cells as well as mouse B16F0 melanoma cells significantly increased the numbers of SA-β-Gal^+^ cells, indicating the induction of tumor cell senescence (Figures 4A,B). However, unlike the effect in colon cancer cells, treatment with baicalein induced more senescent cell populations in both human and mouse melanoma cell lines than that of baicalin treatment in those melanoma cells (Figures 4A,B). Interestingly, we observed that high concentrations of baicalein and baicalin (above 50 μM) were required to induce senescence in B16F0 mouse melanoma cells compared with those in human melanoma cell lines in *in vitro* treatment (Figures 4A,B). These results collectively suggest that baicalein and baicalin treatment in melanoma cells can induce both cell apoptosis and senescence, resulting in the inhibition of tumor cell growth and functions.

### Baicalein and Baicalin Suppress Glucose Metabolism in Melanoma Cells

**FIGURE 5:**
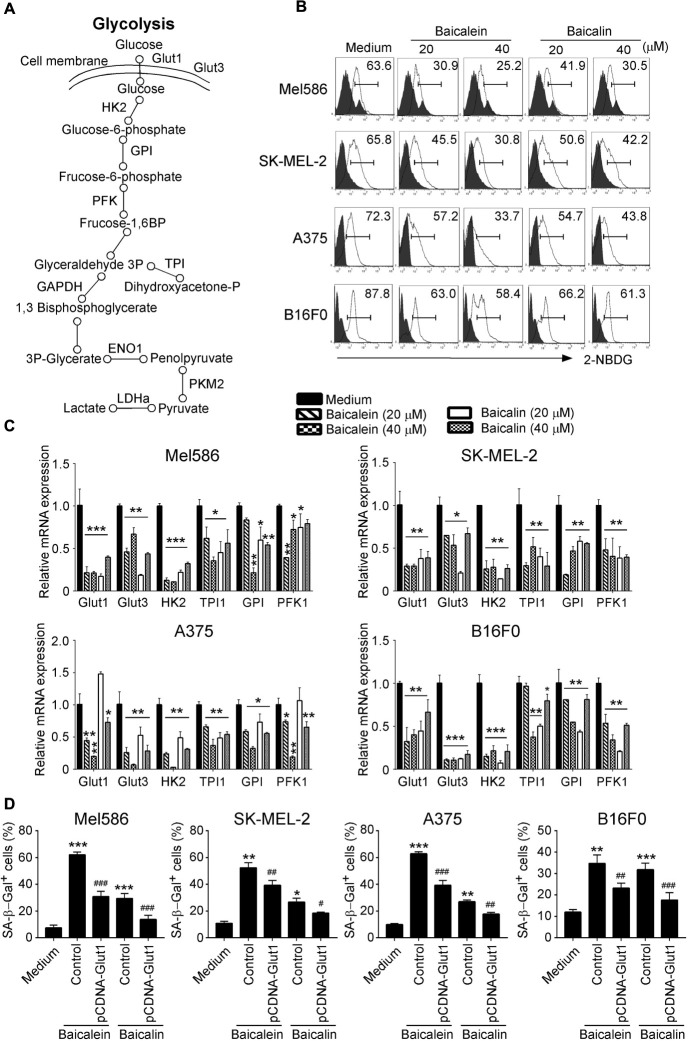
Baicalein and baicalin treatments down-regulate glucose uptake and glycolysis in melanoma cells. **(A)** The sketch map shows the steps and regulations of the key metabolites and enzymes in cell glycolysis. **(B)** Baicalein and baicalin treatments significantly decreased glucose uptake in both human and mouse melanoma cells. Glucose uptake of melanoma cells was determined by the flow cytometry with addition of 2-NBDG (100 μM) for 30 min after 3-day culture with the indicated concentrations of baicalein and baicalin. **(C)** Baicalein and baicalin treatments significantly down-regulated gene expression levels of key glycolytic enzymes in both human and mouse melanoma cells. Melanoma cells were treated with or without indicated concentrations of baicalein and baicalin for 72 h. Total RNA was isolated from the tumor cells and analyzed by real-time PCR. The expression levels of each gene were normalized to β-actin expression levels and adjusted to the levels in untreated tumor cells (medium). Data shown in different melanoma cells are mean ± SD from three independent experiments. *p < 0.05, **p < 0.01, and ***p < 0.001, compared with the medium only group. **(D)** Over-expression of Glut1 prevented cell senescence in melanoma cells induced by baicalein and baicalin. Melanoma cells were transfected with pCDNA-Glut1 or control vector plasmids for 24 h, then cultured for 3 days in the presence of baicalein or baicalin (40 μM for Mel586, A375, and SK-mel-2 cells, and 50 μM for B16F0 cells). Senescent cells were analyzed using the SA-β-Gal activity assay. Data shown are mean ± SD from three independent experiments with similar results. *p < 0.05, **p < 0.01, and ***p < 0.001, compared with the medium control group. ^#^*p* < 0.05, ^##^*p* < 0.01, and ^###^*p* < 0.001, compared with the respective baicalein or baicalin treatment group.

Metabolic dysregulation is one of the characteristic hallmarks of tumor malignancy (Ward and Thompson, 2012; Dang, 2013). Cancer cells often display heightened glucose consumption in the tumor microenvironment. We reasoned that baicalein and baicalin-induced inhibition of tumor proliferation and biological functions may be caused by interfering with cell energy metabolism. We thus investigated the metabolic profile of the melanoma cells treated with baicalein and baicalin (Figure 5A). We first determined the glucose uptake ability of melanoma cells in the presence and absence of different concentrations of baicalein and baicalin, using a fluorescent glucose analog D-glucose analog 2-(N-(7-nitrobenz-2-oxa-1,3-diazol-4-yl) amino)-2-deoxy-D-glucose (2-NBDG) labeling assay (Liu et al., 2018; Li et al., 2019). Our results clearly showed that both human and mouse melanoma cells had high glucose uptake abilities. However, treatments with baicalein and baicalin significantly inhibited glucose uptake abilities of four melanoma cell lines no matter of N-RAS and B-RAF mutation statuses (Figure 5B). We then determined the effect on key glycolytic enzymes in tumor cells using real-time quantitative PCR analyses (Li et al., 2019). Those molecules include glucose transporters 1 and 3 (Glut1 and Glut3), as well as glycolysis-related enzymes hexokinase 2 (HK2), glucose-6-phosphate isomerase (GPI), phosphofructokinase 1 (PFK1), triosephosphate isomerase 1 (TPI1), enolase 1 (ENO1), pyruvate kinase muscle 2 (PKM2), and lactate dehydrogenase A (LDHα) (Figure 5A). Baicalein and baicalin treatments markedly suppressed gene expression of Glut1, Glut3, HK2, TPI, GPI, and PFK1 in both human and mouse melanoma cells (Figure 5C). In addition, both baicalein and baicalin inhibited LDHα expression in Mel586, A375, and B16F0 melanoma cells, and ENO1 expression in SK-MEL-2 and A375 cells, as well as partially suppressed PKM2 expression in SK-MEL-2, A375, and B16F0 tumor cells (Supplementary Figure S2). To further investigate the causative role of glucose metabolism inhibition in cell senescence and suppression of melanoma cells mediated by these two compounds, we determined whether we can prevent tumor cell senescence mediated by baicalein and baicalin if we promote glucose mentalism through overexpression of Glut1 gene in the melanoma cells. Consistent with the above results, baicalein and baicalin treatments significantly increased melanoma cell senescence. However, transfection of pCDNA-Glut1 plasmid but not control vector in melanoma cells dramatically reversed the senescence in the melanoma cells induced by both baicalein and baicalin treatments (Figure 5D). These results collectively suggest that baicalein and baicalin inhibit melanoma tumor growth and function via suppression of tumor cell glucose metabolism.

### Down-Regulation of mTORC1-HIF1α Signaling in Melanoma Cells Is Responsible for Glucose Metabolism Inhibition Induced by Baicalein and Baicalin

**FIGURE 6:**
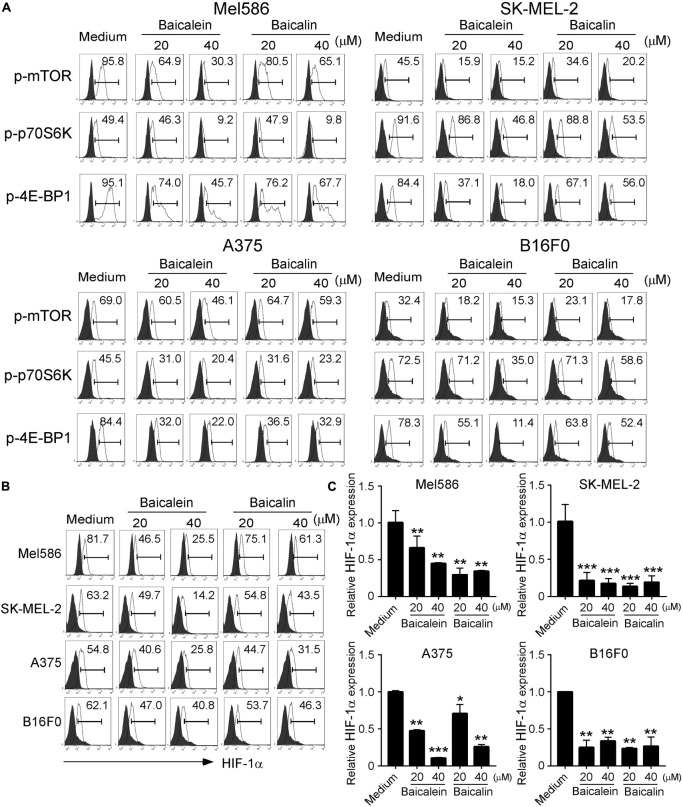
Baicalein and baicalin treatments suppress mTORC1-HIFα signaling in melanoma cells. **(A)** Suppression of phosphorylation and subsequent activation of mTOR signaling in melanoma cells treated with baicalein and baicalin. Melanoma cells were treated with or without indicated concentrations of baicalein and baicalin for 72 h and then phosphorylated mTOR, p70S6K, and 4E-BP1 in tumor cells were determined by the flow cytometry analyses. **(B,C)** Baicalein and baicalin treatments down-regulated HIF1α protein and mRNA expression in melanoma cells. Cell preparation and treatment were identical to panel **(A)**. The protein expression of HIF-1α in treated tumor cells was determined by the flow cytometry **(B)**. Total RNA was isolated from the tumor cells and analyzed by Real-time PCR **(C)**. The gene expression levels of HIF1α were normalized to β-actin expression levels and adjusted to the levels in untreated melanoma cells. Data shown are representatives of mean ± SD from three independent experiments. *p < 0.05, **p < 0.01, and ***p < 0.001, compared with the medium only group.

mTOR signaling pathway plays a central role in metabolic reprogramming of tumor cell growth and proliferation (Sun et al., 2011; Chen et al., 2015). Furthermore, hypoxia-inducible factor 1-alpha (HIF1α) serves as a key transcription factor that performs important functions in regulation of cellular metabolism (Cheng et al., 2014; Pusapati et al., 2016; Salmond, 2018). Our recent studies demonstrated that mTORC1–HIF1α pathway promotes glucose metabolism and glycolysis in Treg cells (Li et al., 2019). We therefore explored the possibility that baicalein and baicalin could inhibit mTORC1–HIF1α signaling pathway and thus result in suppression of glucose metabolism in melanoma cells. To test this possibility, we first analyzed the phosphorylation and activation of mTOR and its downstream substrates p70S6K and 4E-BP1 in melanoma cells treated with or without baicalein and baicalin. We found that both human and mouse melanoma cells have high phosphorylated mTOR and p70S6K cell populations, indicating elevated mTORC1 activity in melanoma tumor cells. However, treatments with baicalein and baicalin significantly suppressed the phosphorylation of mTOR and its downstream substrates p70S6K and 4E-BP1 in the melanoma cell lines, further confirming their inhibition of mTOR signaling in melanoma cells (Figure 6A). Consistent with our above and previous results, baicalein had more potent suppressive effect on mTOR signaling in tumor cells than that of baicalin. To further test the possibility that baicalein and baicalin-mediated suppression of melanoma cells involves the mTORC1–HIF1α signaling pathway regulation, we also determined HIF1α protein and mRNA expression in both human and mouse melanoma cells treated with or without the two compounds using the flow cytometry and Real-time PCR analyses. The four melanoma cell lines expressed high levels of HIF1α. Furthermore, treatments with baicalein and baicalin significantly inhibited HIF1α expression in all melanoma cells, suggesting down-regulation of HIF1α pathway in melanoma cells induced by the two compounds (Figures 6B,C). All these results suggest that baicalein and baicalin down-regulate the mTOR-HIFα signaling in tumor cells.

**FIGURE 7:**
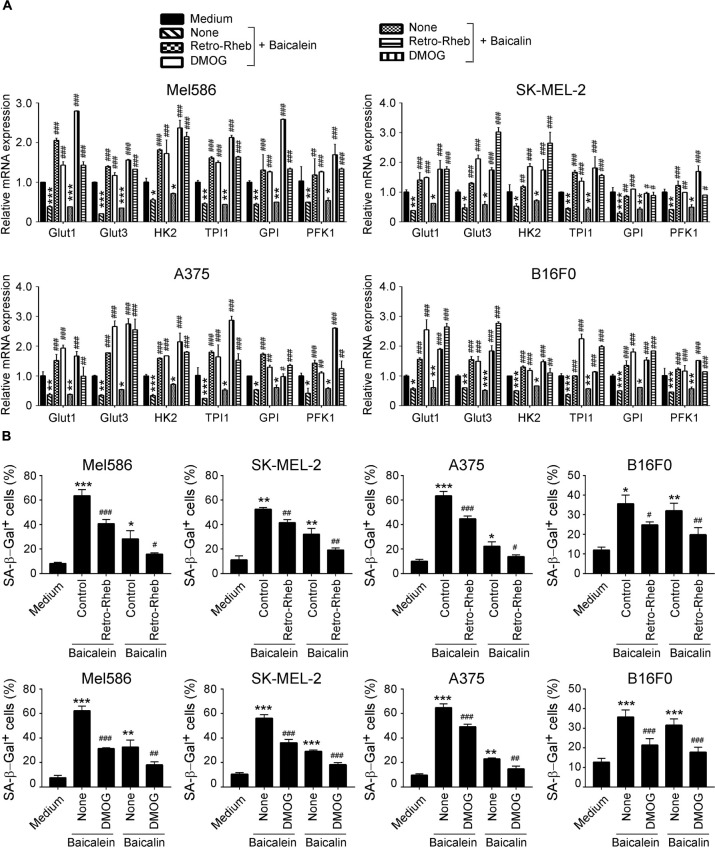
mTORC1-HIFα signaling controls glucose metabolism inhibition and senescence induction in melanoma cells mediated by baicalein and baicalin. **(A)** Activation of mTORC1-HIFα signaling blocked baicalein and baicalin-induced down-regulated gene expression of key glycolytic enzymes in both human and mouse melanoma cells. Melanoma cells were transfected with Retro-Rheb plasmid or pretreated with DMOG (0.1 mM) for 24 h, then cultured for 3 days in the presence of 40 μM of baicalein or baicalin. Total RNA was isolated from the tumor cells and analyzed by Real-time PCR. The expression levels of each gene were normalized to β-actin expression levels and adjusted to the levels in untreated tumor cells (medium). Data shown in different melanoma cells are mean ± SD from three independent experiments. *p < 0.05, **p < 0.01, and ***p < 0.001, compared with the medium control group. ^#^*p* < 0.05, ^##^*p* < 0.01, and ^###^*p* < 0.001, compared with the respective baicalein or baicalin treatment group. **(B)** Activation of mTORC1-HIFα signaling prevented senescence induction in melanoma cells mediated by baicalein and baicalin. Cell treatment and procedure are identical to panel **(A)**. Treated melanoma cells were cultured for 3 days in the presence of baicalein or baicalin (40 μM for Mel586, A375, and SK-MEL-2 cells, and 50 μM for B16F0 cells). Senescent cells were analyzed using the SA-β-Gal activity assay. Data shown are mean ± SD from three independent experiments with similar results. *p < 0.05, **p < 0.01, and ***p < 0.001, compared with the medium control group. ^#^*p* < 0.05, ^##^*p* < 0.01, and ^###^*p* < 0.001, compared with the respective baicalein or baicalin treatment group.

We then confirmed the functional importance of mTOR-HIFα signaling pathway in glucose metabolism suppression and senescence induction in melanoma cells mediated by baicalein and baicalin. We performed a functional rescue experiment with over-expression of the mTOR upstream activator Rheb gene in melanoma cells (retrovirus-based Rheb) (Yang et al., 2017; Li et al., 2019). Activation of mTOR signaling with Retro-Rheb transfection significantly reversed suppression of gene expression levels of glucose transporters and glycolytic enzymes in melanoma cells induced by baicalein and baicalin (Figure 7A). Furthermore, mTOR signaling activation markedly prevented the baicalein and baicalin-induced senescence in both human and mouse melanoma cells (Figure 7B). In addition, we activated HIF1α function with the specific pharmacological activator dimethyloxalylglycine (DMOG) and then evaluated glycolysis inhibition and cell senescence induction mediated by baicalein and baicalin (Zhdanov et al., 2015; Cosin-Roger et al., 2017). Consistent with the results from Retro-Rheb transfection, activation of HIF1α signaling with DMOG in tumor cells markedly blocked the inhibition of gene expression of glucose transporters and glycolytic enzymes and prevented induction of senescence in melanoma cells mediated by baicalein and baicalin (Figures 7A,B). These results indicate that mTOR-HIF1α axis and its downstream glycolytic program is critical and involved in tumor growth suppression and senescence induction in melanoma cells mediated by baicalein and baicalin.

### Baicalein and Baicalin Inhibit Tumorigenesis and Growth of Melanoma *in vivo*

**FIGURE 8:**
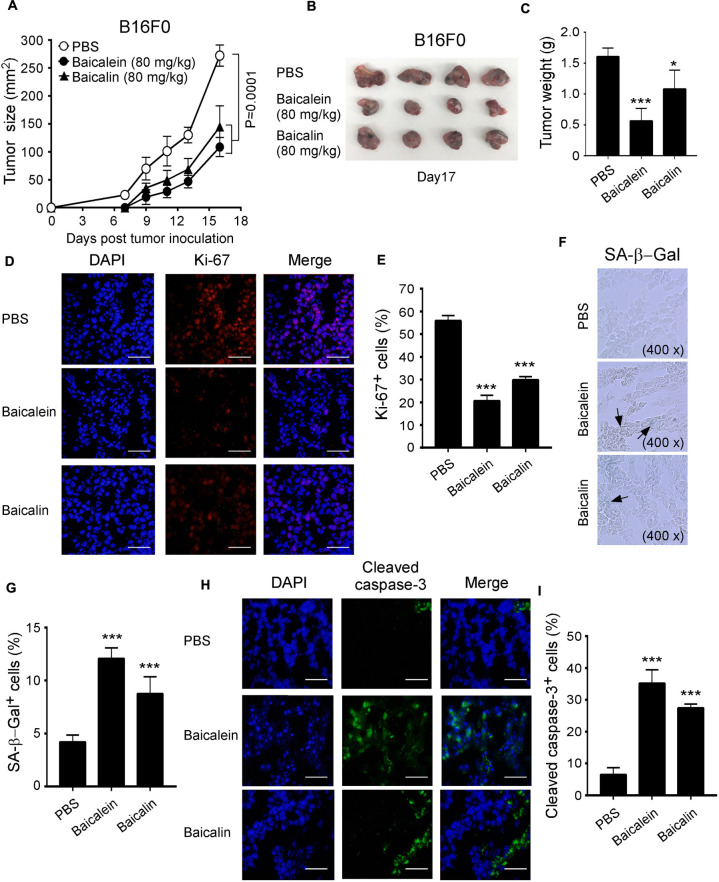
Baicalein and baicalin inhibit tumor growth and tumorigenesis of melanoma *in vivo*. **(A)** Both baicalein and baicalin dramatically inhibited B16F0 melanoma tumor growth in NSG immunodeficient mice. B16F0 cells (1 × 10^5/^mouse) were subcutaneously injected into NSG mice. After 4 days post tumor injection (tumor size reached around 5 × 5 mm), the tumor-bearing mice were administrated with baicalein (80 mg/kg), baicalin (80 mg/kg), or PBS control through intraperitoneal injection, respectively at every other day for 2 weeks. Tumor volumes were measured and presented as mean ± SD (*n* = 4 mice/group). *p* values were determined by the one-way analysis of variance (ANOVA). **(B)** Representative image of B16F0 tumors obtained from the indicated groups at the endpoint of the experiments (day 17). **(C)** Treatments with both baicalein and baicalin had much lower tumor weights compared with that of PBS control group. Results shown are mean ± SD of the tumor weights from the indicated groups in the B16F0 model at the endpoint of the experiments (day 17) (*n* = 4 mice/group). *p < 0.05 and ***p < 0.001, compared with the PBS injection group using unpaired *t*-test. **(D,E)** Treatments with baicalein and baicalin significantly decreased the Ki-67^+^ cell populations in tumor tissues at the endpoint of experiment using an immunofluorescence assay. Panel **(D)** are representative images of Ki-67 expression in tumor tissues from different groups. Scale bar: 50 μm. Panel **(E)** is the summary of mean ± SD of KI-67^+^ cell fractions per high microscope field (×400) in the tumor tissues from four mice of each group. ***p < 0.001, compared with the PBS control treatment mice using unpaired *t*-test. **(F,G)** Large amounts of senescent tumor cells were observed in the tumor tissues from both treatments of baicalein and baicalin in NSG mice. SA-β-Gal expression was determined in the tumor frozen tissues from different groups at the endpoint of experiment. Panel **(F)** is photomicrographs of SA-β-Gal expression in tumor tissues from different groups as arrows indicated. Panel **(G)** is the summary of mean ± SD of SA-β-Gal^+^ cell numbers per high microscope field (×400) in the tumor tissues from four mice of each group. ***p < 0.001, compared with the PBS treatment control mice using unpaired *t*-test. **(H,I)** Increased apoptotic cells were observed in B16F0 tumor tissues from the treatment groups with baicalein and baicalin. Cell apoptosis in the frozen sections was analyzed by the anti-cleaved caspase-3 staining at the end of experiments. Panel **(H)** are representative images of cleaved caspase-3 expression in tumor tissues from different groups. Scale bar: 50 μm. Panel **(I)** is the summary of mean ± SD of cleaved caspase-3^+^ cell fractions per high microscope field (×400) in the tumor tissues from four mice of each group. ***p < 0.001, compared with the PBS treatment control group using unpaired *t*-test.

Our *in vitro* studies have clearly demonstrated that baicalein and baicalin could suppress glucose metabolism in melanoma cells, resulting in suppression of tumor cell growth and functions. We next performed complementary *in vivo* studies using mouse melanoma B16F0 cells in the humanized NOD-scid IL2Rγ^*null*^ (NSG) mouse xenograft models, and explored whether baicalein and baicalin can inhibit tumorigenesis and growth of melanoma *in vivo*. B16F0 melanoma cells were subcutaneously injected into NSG mice. After 4 days post tumor injection (tumor size reached around 5 × 5 mm), baicalein and baicalin (80 mg/kg) were administered through intraperitoneal injection into the tumor-bearing mice, respectively, at every other day for 2 weeks. Tumor growth was evaluated. At the end of experiments (day 17), tumors were isolated from the different groups of the sacrificed mice and weighted. B16F0 tumor cells injected with PBS control grew progressively in NSG mice. However, treatments with both baicalein and baicalin significantly inhibited tumor growth (Figure 8A). Furthermore, tumor sizes collected from the baicalein- or baicalin-treated B16F0 groups were much smaller than those in the PBS treatment group (Figure 8B). In addition, the average tumor weights obtained from either the baicalein treatment group or baicalin treatment group were much lower than that of control group (Figure 8C). Notably, consistent with the results from *in vitro* studies, baicalein had more potent anti-tumor effect than that of baicalin *in vivo*.

In addition to the tumor growth evaluation, we investigated the tumor cell proliferation, senescence and apoptosis mediated by baicalein and baicalin treatments in tumor tissues from different groups. We observed that baicalein and baicalin treatments markedly suppressed tumor cell proliferation as indicated by a decrease of Ki-67^+^ cell populations in tumor tissues (Figures 8D,E). Furthermore, baicalein and baicalin treatments also significantly induced increases of both senescent and apoptotic cell populations in tumor tissues as determined with SA-β-Gal and cleaved caspase-3 staining analyses, respectively (Figures 8F–I). All these studies collectively indicate that baicalein and baicalin can induce tumor cell senescence and apoptosis, and suppress tumor proliferation and growth *in vivo*. We then further confirmed whether baicalein and baicalin-mediated inhibition of tumor growth *in vivo* is due to suppression of glucose metabolism in tumor cells. We detected the gene expression of the key enzymes involved in glycolysis in tumor tissues obtained from different groups. We observed significantly decreased gene expression levels of glucose transporters Glut1 and Glut3, as well as the key glycolytic enzymes in tumor tissues treated with baicalein or baicalin, further suggesting inhibition of glycolysis in tumor cells (Supplementary Figure S3). Collectively, our studies clearly indicate that baicalein and baicalin can suppress tumor cell metabolism, promote cell senescence and apoptosis, and inhibit tumor proliferation and growth *in vitro* and *in vivo*.

## Discussion

Melanoma is one of the most common cancers worldwide and has a high rate of death with the advanced disease. Development of novel therapeutic strategies and/or effective drugs is a significant challenge and urgently needed for clinical patients (Brose et al., 2002; Omholt et al., 2003; Thumar et al., 2014). Our current studies have identified that the Chinese traditional medicines baicalein and baicalin are potent anti-tumor agents for melanoma even with N-RAS and B-RAF mutations. Baicalein and baicalin strongly inhibit melanoma cell behaviors and functions, including tumor cell growth and proliferation, as well as colony formation and migration. Mechanistic studies further demonstrated that baicalein and baicalin-mediated suppression of melanoma growth and development is through the inhibition of glucose metabolism in tumor cells, promoting tumor cell apoptosis and senescence. Our studies indicate that baicalein and baicalin could be potentially novel and effective therapeutic drugs for melanoma treatment.

Although significant progress has been made to develop target therapy for melanoma, the mutations on N-RAS and B-RAF occurred in tumor cells have been a significant challenge for successful treatment of melanoma patients (Brose et al., 2002; Omholt et al., 2003; Thumar et al., 2014). Patients with N-RAS and B-RAF mutations have higher incidences of central nervous system (CNS) metastasis in stage IV disease than those with wild-type B-RAF and N-RAS (Flanigan et al., 2013). Furthermore, N-RAS mutation status has been proved to be associated with shorter survival from stage IV melanoma patients and be an independent predictor for clinical outcomes (Ugurel et al., 2007; Jakob et al., 2012). Although many inhibitors have been developed to target the mutations and/or activated signaling pathways, the overall effects of the inhibitors against melanoma are limited in certain types of clinical patients (Brose et al., 2002; Thumar et al., 2014). Moreover, resistance against the target inhibitors and/or activation of alternative survival signaling pathways in cancer cells also keep merging in cancer patients (Nazarian et al., 2010; Villanueva et al., 2010; Gowrishankar et al., 2012). In addition, the currently developed inhibitors, such as PLX4720 and PLX4032, show limitations as they could not cure melanoma with both N-RAS and B-RAF mutations (Halaban et al., 2010; Kaplan et al., 2012). These are significantly obstacles for a target therapy against melanoma. Therefore, development of alternative new drugs which can target melanoma cells with or without different types of oncogenic mutations is needed. In fact, many promising drugs have been developed based on the natural products and applied in clinical trials for various disease treatments including cancers (Cragg et al., 2009; Cragg and Newman, 2013; Stover et al., 2014). In our current studies, we utilized different types of melanoma cell lines with/without N-RAS and B-RAF mutations, including Mel586, SK-MEL-2 (wild type B-Raf and mutant N-RAS), A375 (B-RAF V600E and wild type N-RAS), as well as mouse B16F0 melanoma cell line. Our results clearly demonstrated that baicalein and baicalin can significantly inhibit both human and mouse melanoma cancer cell growth and proliferation regardless of N-RAS and B-RAF mutation statuses in tumor cells. In addition, the suppression of melanoma cells is due to the promotion of cancer cell apoptosis and senescence mediated by baicalein and baicalin. All these studies indicate that baicalein and baicalin could be promising and effective drugs targeting different mutants for melanoma therapy.

*Scutellaria baicalensis* Georgi is one of the most important traditional Chinese medicines, which is widely used for the disease treatments. Baicalein and baicalin are active components of *Scutellaria baicalensis* Georgi. Increasing evidence suggests that baicalein and baicalin have strong anti-tumor effects in various cancers (Takahashi et al., 2011; Huang et al., 2012; Zhang et al., 2013; Aryal et al., 2014; Chung et al., 2015). Our previous studies have further demonstrated that baicalein and baicalin can suppress colon cancer cell proliferation and growth *in vitro* and *in vivo* (Dou et al., 2018; Wang et al., 2018). Thus, exploring anti-tumor efficacy in different cancer types and related mechanisms mediated by baicalein and baicalin will provide critical information for the development of novel strategies for cancer treatment. We have demonstrated that MAPK ERK and p38 signaling pathways are involved in baicalein and baicalin-induced apoptosis and senescence in colon cancer (Dou et al., 2018). Furthermore, we identified that baicalin up-regulates the expression of DEPP (progesterone) and activates its downstream Ras/Raf/MEK/ERK and p16INK4A/Rb signaling pathways by acting as an antioxidant, leading to senescence in colon cancer cells (Wang et al., 2018). In this study, we provide first evidence that baicalein and baicalin can induce melanoma cell apoptosis and senescence, which is consistent with our previous studies shown in colon cancer (Dou et al., 2018; Wang et al., 2018). Importantly, our current studies further identified the metabolic control as a novel molecular mechanism responsible for the tumor suppression mediated by baicalein and baicalin. Increasing evidence suggests that metabolic disorder is a significant hallmark in the malignant tumors which controls the progression of tumor biological behaviors and immune cell functions in the suppressive tumor microenvironment (Biswas, 2015; Yoshida, 2015). Furthermore, the “Warburg effect” has been widely accepted as a common feature of metabolic reprogramming in tumors, and tumor cells depend on aerobic glycolysis for maintaining biosynthesis and functions (Warburg, 1956; Koppenol et al., 2011). In our effort to identify how baicalein and baicalin molecularly inhibit melanoma growth, we demonstrated that baicalein and baicalin can metabolically reprogram melanoma cells via inhibition of glucose uptake and downregulation of the key enzymes in glucose transport and glycolysis in tumor cells. Furthermore, the metabolic inhibition by baicalein and baicalin involves the mTOR-HIF-1α signaling regulation. All the results from both *in vivo* and *in vitro* studies indicate that baicalein and baicalin can rewrite tumor metabolism and have potent anti-tumor effects in melanoma. We will continue our future studies to explore the possibility that combining baicalein or baicalin treatment with other therapeutic strategies including immunotherapy will synergistically enhance anti-tumor efficacy in different melanoma models.

In summary, we report that baicalein and baicalin can significantly inhibit melanoma regardless of mutation statuses *in vitro* and *in vivo*. We further revealed that their anti-tumor effects are mechanistically due to the suppression of cancer cell glucose metabolism and induction of melanoma cell apoptosis and senescence. These data clearly suggest that both baicalein and baicalin have potent anti-tumor effects against melanoma and are potential novel and universal target drugs for melanoma therapy.

## Materials and Methods

### Chemical Compounds

Baicalein (Purity 98.5%) and baicalin (Purity 91.5%) were purchased from the Kanghua Company (Nanjing, Jiangsu, China) and were dissolved in dimethyl sulfoxide (DMSO, Sigma, St. Louis, Mo, United States). A 50 mM stock solution were prepared and stored in −20°C for the experiments, as we previously described (Dou et al., 2018; Wang et al., 2018).

### Melanoma Cell Lines

Human melanoma cell lines (A375 and SK-MEL-2) and mouse melanoma cell line B16F were originally purchased from the American Type Culture Collection (ATCC, Manassas, VA, United States). Human Mel586 were obtained from the National Cancer Institute (NCI). Mel586 and B16F0 cells were maintained in RPMI-1640 medium containing 10% fetal bovine serum (FBS). A375 and SK-MEL-2 were maintained in DMEM medium containing 10% FBS.

### Cell Growth and Proliferation Assay

Melanoma cell lines were cultured at a started number of 2 × 10^5^/well in 6-well plates in the presence of different concentrations of baicalein and baicalin in triplicate wells, as we previously described (Dou et al., 2018; Wang et al., 2018). Cell growth was evaluated at different time points using the cell number counting. In addition, cell proliferation was determined using the [^3^H]-thymidine incorporation assays as we previously described (Ye et al., 2012, 2014). In brief, different numbers of tumor cells (5 × 10^3^, 1 × 10^4^ or 2 × 10^4^) were cultured in 96-well plates in cell assay medium containing 2% FCS in the presence of different concentrations of baicalein and baicalin. After 56 h of culture, [^3^H]-thymidine was added at a final concentration of 1 μCi/well, followed by an additional 16 h of culture. The incorporation of [^3^H]-thymidine was measured with a liquid scintillation counter (PerkinElmer, Waltham, MA, United States).

### Colony Formation Assay

Two hundred to five hundred per well of melanoma cells treated with different concentrations of baicalein or baicalin, were seeded in 6-well plates and cultured for 10–14 days. Cell colonies were fixed with 4% formaldehyde, stained with 0.5% crystal violet for 15 min at room temperature, washed for several times and then counted under a microscope, as we described previously (Liu et al., 2015; Dou et al., 2018; Wang et al., 2018).

### Wound Healing Assay

Melanoma cells were plated in 6-well plates and grown to 60–80% confluence. A wound area was generated by scraping cells with a 10 μl pipette tip across the entire diameter of the dish and extensively rinsed with PBS to remove all cellular debris. The scratches were photographed after additional 24 and 36 h of culture in the presence of different concentrations of baicalein or baicalin. The closure was estimated as the wounded area relative to the initial area (Liu et al., 2015; Dou et al., 2018; Wang et al., 2018).

### Adhesion Assay

The flat bottom 96-well plates were coated with fibronectin (10 μl/ml, BD Biosciences) at 4°C overnight and then blocked with 2% BSA in PBS at 37°C for 2 h. Melanoma cells (1 × 10^5^/ml) pre-treated with different concentrations of baicalein or baicalin for 72 h were seeded into fibronectin-pre-coated 96-well plates (10 μl/well) in the medium without FBS and incubated for 45 min. After washing three times with PBS to remove non-adherent cells, the cells attached on the plates were fixed with 4% formaldehyde for 4 min and stained with 0.03% crystal violet for 15 min. Adherent cells were counted and averaged in 10 fields at a high (×400) magnification with a microscope (Liu et al., 2015).

### Apoptosis Assays

Melanoma cells were cultured for 36 or 72 h in the presence of different concentrations of baicalein and baicalin, and apoptosis was analyzed after staining with PE-labeled Annexin V and 7-AAD (BD Biosciences, San Diego, CA, United States) (Liu et al., 2015; Dou et al., 2018; Wang et al., 2018). Stained cells were analyzed on a FACSCalibur (BD Bioscience) and the data were analyzed with the FlowJo software (Tree Star, Ashland, OR, United States).

### Flow Cytometry Analysis

The expression of mTOR-HIF-1α signaling markers on tumor cells were determined by FACS analysis after staining with anti-human specific antibodies, including anti-phosphorylated mTOR (1:500), p70S6K (1:500), and 4E-BP1 (1:1000), as well as anti-HIF-1α (1:1000) and then secondary anti-rabbit antibody conjugated with either PE or FITC. These antibodies were purchased from Cell Signaling Technology. All stained cells were analyzed on a FACSCalibur flow cytometer (BD Bioscience) and data analyzed with the FlowJo software (Tree Star).

### Senescence Associated β-Galactosidase (SA-β-Gal) Staining

Senescence associated β-Galactosidase (SA-β-Gal) activity in tumor cells was detected as we previously described (Dou et al., 2018; Wang et al., 2018). Briefly, melanoma cells were cultured for 3 days in the presence of different concentrations of baicalein or baicalin. For some experiments, tumor cells were transfected with Retro-Rheb, pCDNA-Glut1 or control plasmids, or pretreated with DMOG (0.1 mM, Sigma) for 24 h, then cultured for 3 days in the presence of different concentrations of baicalein or baicalin (Li et al., 2019). Tumor cells were fixed in 3% formaldehyde, and followed to incubate overnight at 37°C with freshly prepared SA-β-Gal staining solution. The stained cells were washed with PBS and examined with a microscope.

### Quantitative Real-Time PCR Analysis

Total RNA was extracted from the mouse and human melanoma cells using the Trizol reagent (Invitrogen), and cDNA was transcribed using a SuperScript II RT kit (Invitrogen), both according to the manufacturers’ instructions. Expression levels of each gene were determined by reverse-transcription PCR using specific primers, and mRNA levels in each sample were normalized to the relative quantity of β-actin gene expression. All experiments were performed in triplicate. The specific primers used for mouse and human metabolic genes are listed in Supplementary Table S1. All primers were purchased from Integrated DNA Technologies.

### Glucose Uptake Assay

Glucose uptake was determined following 20 min incubation of melanoma cells with a fluorescent D-glucose analog 2-[(7-nitrobenz-2-oxa-1,3-diazol-4-yl) amino]-2-deoxy-D-glucose (2-NBDG) (Cayman Chemical), as we previously described (Li et al., 2019). Melanoma cells were pretreated with different concentrations of baicalein and baicalin for 72 h. Treated and untreated melanoma cells were cultured in the glucose-free medium for 30 min, and followed addition of 2-NBDG (100 μM) for 20 min and analyzed with a FACSCalibur flow cytometer (BD Bioscience).

### Immunofluorescence Staining in Tissues

Tumor tissues were embedded into OCT and prepared for cryostat sections (4∼8 μm). Frozen slides were recovered to room temperature, washed with PBS and treated with 3% H_2_O_2_ in PBS for 30 min. The sections were further blocked with 3.7% formaldehyde for 30 min and added the primary antibodies, including anti-Ki-67 (#9129, Cell Signaling Technology), anti-cleaved caspase-3 (#9664, Cell Signaling Technology) at diluted concentrations of 1:50 and 1:400, respectively, under 4°C overnight. The slides were washed with PBS and added a secondary antibody-conjugated with AF594 and DAPI at diluted concentrations of 1:300 and 1:2500, under room temperature for 1 h. The stained slides were analyzed by an immunofluorescence microscopy.

### *In vivo* Tumorigenesis Studies

NOD-scid IL2Rγ^*null*^ (NSG, 6–8 weeks) immunodeficient mice were purchased from The Jackson Laboratory and maintained in the institutional animal facility. All animal studies have been approved by the Institutional Animal Care Committee. For tumorigenesis studies, B16F0 cells (1 × 10^5^/mouse) were subcutaneously injected into NSG mice. After 4 days post tumor injection (tumor size reached around 5 × 5 mm), the tumor-bearing mice were randomly divided into three groups (*n* = 4/group) and administrated with baicalein (80 mg/kg), baicalin (80 mg/kg), and PBS control through intraperitoneal injection, respectively, at every other day for 2 weeks. Tumor size was measured with calipers every 2 days. Tumor volume was calculated on the basis of two-dimensional measurements. At the end of experiments (day 17), the mice were sacrificed and tumors were isolated and weighted. Furthermore, tumor tissues were embedded into OCT and prepared for cryostat sections (4∼8 μm), and SA-β-Gal expression, cell proliferation and apoptotic cell populations were assayed, as described above. In addition, parts of tumor issues were grinded and total RNA was extracted, and cDNA was transcribed for RT-PCR experiments.

### Statistical Analysis

Statistical analysis was performed with GraphPad Prism5 software. Data are expressed as mean ± standard deviation (SD). For multiple group comparison *in vivo* studies, the one-way analysis of variance (ANOVA) was used, followed by the Dunnett’s test for comparing experimental groups against a single control. For single comparison between two groups, paired Student’s *t*-test was used. Non-parametric *t*-test was chosen if the sample size was too small and did not fit a Gaussian distribution.

## Data Availability Statement

The raw data supporting the conclusions of this article will be made available by the authors, without undue reservation.

## Ethics Statement

The animal study was reviewed and approved by the Saint Louis University.

## Author Contributions

LH, HX, and GP designed research, analyzed the data, prepared figures, and wrote the manuscript. LH, BP, YN, CW, FS, and XL performed the experiments. JD provided the baicalein and baicalin compounds and discussion of the manuscript. All authors contributed to the article and approved the submitted version.

## Conflict of Interest

The authors declare that the research was conducted in the absence of any commercial or financial relationships that could be construed as a potential conflict of interest.

## Supplementary Material

FIGURE S1 Increased apoptotic cell populations are induced in both human and mouse melanoma cells after treatments with baicalein and baicalin. Tumor cells were cultured in the presence of the indicated concentrations of baicalein and baicalin for 36 h. Apoptosis in treated tumor cells was analyzed after staining with PE-labeled Annexin V and 7-AAD. Results shown in the histogram are summaries of mean ± SD from three independent experiments. ^∗∗∗^*p* < 0.001, compared with the medium control group.

FIGURE S2 Baicalein and baicalin treatments significantly down-regulate gene expression levels of key glycolytic enzymes in melanoma cells. Both human and mouse Melanoma cells were treated with or without the indicated concentrations of baicalein and baicalin for 72 h. Total RNA was isolated from the tumor cells and analyzed by Real-time PCR. The expression levels of each gene were normalized to β-actin expression levels and adjusted to the levels in untreated tumor cells (medium). Data shown in different melanoma cells are mean ± SD from three independent experiments. ^∗^*p* < 0.05 and ^∗∗^*p* < 0.01, compared with the medium only group.

FIGURE S3 Baicalein and baicalin treatments down-regulate gene expression levels of key glycolytic enzymes in B16F0 tumor cells *in vivo*. B16F0 cells (1 × 10^5/^mouse) were subcutaneously injected into NSG mice. After 4 days post tumor injection (tumor size reached around 5 × 5 mm), the tumor-bearing mice were administrated with baicalein (80 mg/kg), baicalin (80 mg/kg), or PBS control through intraperitoneal injection, respectively at every other day for 2 weeks. At the end of experiments, tumor issues were grinded and total RNA was extracted. The expression levels of each gene were normalized to β-actin expression levels and adjusted to the levels in PBS treated group (set as 1). Results shown are mean ± SD from four mice per group. ^∗^*p* < 0.05, ^∗∗^*p* < 0.01, and ^∗∗∗^*p* < 0.001, compared with the PBS treatment control group using unpaired *t*-test.

## References

[B1] American Cancer Society (2018). *Cancer Facts and Figures 2018.* Atlanta, GA: American Cancer Society.

[B2] Aryal P. Kim K. Park P. H. Ham S. Cho J. Song K. (2014). Baicalein induces autophagic cell death through AMPK/ULK1 activation and downregulation of mTORC1 complex components in human cancer cells. *FEBS J.* 281 4644–4658. 10.1111/febs.12969 25132405

[B3] Biswas S. K. (2015). Metabolic reprogramming of immune cells in cancer progression. *Immunity* 43 435–449. 10.1016/j.immuni.2015.09.001 26377897

[B4] Brose M. S. Volpe P. Feldman M. Kumar M. Rishi I. Gerrero R. (2002). BRAF and RAS mutations in human lung cancer and melanoma. *Cancer Res.* 62 6997–7000. 12460918

[B5] Chao J. I. Su W. C. Liu H. F. (2007). Baicalein induces cancer cell death and proliferation retardation by the inhibition of CDC2 kinase and survivin associated with opposite role of p38 mitogen-activated protein kinase and AKT. *Mol. Cancer Ther.* 6 3039–3048. 10.1158/1535-7163.mct-07-0281 18025287

[B6] Chen G. Q. Tang C. F. Shi X. K. Lin C. Y. Fatima S. Pan X. H. (2015). Halofuginone inhibits colorectal cancer growth through suppression of Akt/mTORC1 signaling and glucose metabolism. *Oncotarget* 6 24148–24162. 10.18632/oncotarget.4376 26160839 PMC4695176

[B7] Cheng S. C. Quintin J. Cramer R. A. Shepardson K. M. Saeed S. Kumar V. (2014). mTOR- and HIF-1alpha-mediated aerobic glycolysis as metabolic basis for trained immunity. *Science* 345:1250684. 10.1126/science.1250684 25258083 PMC4226238

[B8] Chiu Y. W. Lin T. H. Huang W. S. Teng C. Y. Liou Y. S. Kuo W. H. (2011). Baicalein inhibits the migration and invasive properties of human hepatoma cells. *Toxicol. Appl. Pharmacol.* 255 316–326. 10.1016/j.taap.2011.07.008 21803068

[B9] Chung H. Choi H. S. Seo E. K. Kang D. H. Oh E. S. (2015). Baicalin and baicalein inhibit transforming growth factor-beta1-mediated epithelial-mesenchymal transition in human breast epithelial cells. *Biochem. Biophys. Res. Commun.* 458 707–713. 10.1016/j.bbrc.2015.02.032 25686495

[B10] Cosin-Roger J. Simmen S. Melhem H. Atrott K. Frey-Wagner I. Hausmann M. (2017). Hypoxia ameliorates intestinal inflammation through NLRP3/mTOR downregulation and autophagy activation. *Nat. Commun.* 8:98. 10.1038/s41467-017-00213-3 PMC5524634 28740109

[B11] Cragg G. M. Grothaus P. G. Newman D. J. (2009). Impact of natural products on developing new anti-cancer agents. *Chem. Rev.* 109 3012–3043. 10.1021/cr900019j 19422222

[B12] Cragg G. M. Newman D. J. (2013). Natural products: a continuing source of novel drug leads. *Biochim. Biophys. Acta* 1830 3670–3695. 10.1016/j.bbagen.2013.02.008 23428572 PMC3672862

[B13] Dang C. V. (2013). MYC, metabolism, cell growth, and tumorigenesis. *Cold Spring Harb. Perspect. Med.* 3:a014217. 10.1101/cshperspect.a014217 23906881 PMC3721271

[B14] de Oliveira M. R. Nabavi S. F. Habtemariam S. Erdogan Orhan I. Daglia M. Nabavi S. M. (2015). The effects of baicalein and baicalin on mitochondrial function and dynamics: a review. *Pharmacol. Res.* 100 296–308. 10.1016/j.phrs.2015.08.021 26318266

[B15] Ding Y. Dou J. Teng Z. Yu J. Wang T. Lu N. (2014). Antiviral activity of baicalin against influenza A (H1N1/H3N2) virus in cell culture and in mice and its inhibition of neuraminidase. *Arch. Virol.* 159 3269–3278. 10.1007/s00705-014-2192-2 25078390

[B16] Dou J. Wang Z. Ma L. Peng B. Mao K. Li C. (2018). Baicalein and baicalin inhibit colon cancer using two distinct fashions of apoptosis and senescence. *Oncotarget* 9 20089–20102. 10.18632/oncotarget.24015 29732005 PMC5929448

[B17] Flaherty K. T. Puzanov I. Kim K. B. Ribas A. McArthur G. A. Sosman J. A. (2010). Inhibition of mutated, activated BRAF in metastatic melanoma. *N. Engl. J. Med.* 363 809–819. 20818844 10.1056/NEJMoa1002011 PMC3724529

[B18] Flanigan J. C. Jilaveanu L. B. Chiang V. L. Kluger H. M. (2013). Advances in therapy for melanoma brain metastases. *Clin. Dermatol.* 31 264–281. 10.1016/j.clindermatol.2012.08.008 23608446

[B19] Gong W. Y. Zhao Z. X. Liu B. J. Lu L. W. Dong J. C. (2017). Exploring the chemopreventive properties and perspectives of baicalin and its aglycone baicalein in solid tumors. *Eur. J. Med. Chem.* 126 844–852. 10.1016/j.ejmech.2016.11.058 27960146

[B20] Gowrishankar K. Snoyman S. Pupo G. M. Becker T. M. Kefford R. F. Rizos H. (2012). Acquired resistance to BRAF inhibition can confer cross-resistance to combined BRAF/MEK inhibition. *J. Invest. Dermatol.* 132 1850–1859. 10.1038/jid.2012.63 22437314

[B21] Halaban R. Zhang W. Bacchiocchi A. Cheng E. Parisi F. Ariyan S. (2010). PLX4032, a selective BRAF(V600E) kinase inhibitor, activates the ERK pathway and enhances cell migration and proliferation of BRAF melanoma cells. *Pigment Cell Melanoma Res.* 23 190–200. 10.1111/j.1755-148x.2010.00685.x 20149136 PMC2848976

[B22] Hatzivassiliou G. Song K. Yen I. Brandhuber B. J. Anderson D. J. Alvarado R. (2010). RAF inhibitors prime wild-type RAF to activate the MAPK pathway and enhance growth. *Nature* 464 431–435. 10.1038/nature08833 20130576

[B23] Huang Y. Hu J. Zheng J. Li J. Wei T. Zheng Z. (2012). Down-regulation of the PI3K/Akt signaling pathway and induction of apoptosis in CA46 Burkitt lymphoma cells by baicalin. *J. Exp. Clin. Cancer Res.* 31:48. 10.1186/1756-9966-31-48 PMC3403945 22607709

[B24] Jakob J. A. Bassett R. L. Jr. Ng C. S. Curry J. L. Joseph R. W. Alvarado G. C. (2012). NRAS mutation status is an independent prognostic factor in metastatic melanoma. *Cancer* 118 4014–4023. 10.1002/cncr.26724 22180178 PMC3310961

[B25] Ji S. Li R. Wang Q. Miao W. J. Li Z. W. Si L. L. (2015). Anti-H1N1 virus, cytotoxic and Nrf2 activation activities of chemical constituents from Scutellaria baicalensis. *J. Ethnopharmacol.* 176 475–484. 10.1016/j.jep.2015.11.018 26578185

[B26] Johnson J. J. (2011). Carnosol: a promising anti-cancer and anti-inflammatory agent. *Cancer Lett.* 305 1–7. 10.1016/j.canlet.2011.02.005 21382660 PMC3070765

[B27] Kaplan F. M. Kugel C. H. III Dadpey N. Shao Y. Abel E. V. Aplin A. E. (2012). SHOC2 and CRAF mediate ERK1/2 reactivation in mutant NRAS-mediated resistance to RAF inhibitor. *J. Biol. Chem.* 287 41797–41807. 10.1074/jbc.m112.390906 23076151 PMC3516728

[B28] Kaplan F. M. Shao Y. Mayberry M. M. Aplin A. E. (2011). Hyperactivation of MEK-ERK1/2 signaling and resistance to apoptosis induced by the oncogenic B-RAF inhibitor, PLX4720, in mutant N-RAS melanoma cells. *Oncogene* 30 366–371. 10.1038/onc.2010.408 20818433 PMC6591715

[B29] Koppenol W. H. Bounds P. L. Dang C. V. (2011). Otto Warburg’s contributions to current concepts of cancer metabolism. *Nat. Rev. Cancer* 11 325–337. 10.1038/nrc3038 21508971

[B30] Kwong L. N. Costello J. C. Liu H. Jiang S. Helms T. L. Langsdorf A. E. (2012). Oncogenic NRAS signaling differentially regulates survival and proliferation in melanoma. *Nat. Med.* 18 1503–1510. 10.1038/nm.2941 22983396 PMC3777533

[B31] Li L. Liu X. Sanders K. L. Edwards J. L. Ye J. Si F. (2019). TLR8-mediated metabolic control of human treg function: a mechanistic target for cancer immunotherapy. *Cell Metab.* 29 103–123. 30344014 10.1016/j.cmet.2018.09.020 PMC7050437

[B32] Liu S. Han B. Zhang Q. Dou J. Wang F. Lin W. (2015). Vasohibin-1 suppresses colon cancer. *Oncotarget* 6 7880–7898. 10.18632/oncotarget.3493 25797264 PMC4480723

[B33] Liu X. Mo W. Ye J. Li L. Zhang Y. Hsueh E. C. (2018). Regulatory T cells trigger effector T cell DNA damage and senescence caused by metabolic competition. *Nat. Commun.* 9:249. 10.1038/s41467-017-02689-5 PMC5770447 29339767

[B34] Moghaddam E. Teoh B. T. Sam S. S. Lani R. Hassandarvish P. Chik Z. (2014). Baicalin, a metabolite of baicalein with antiviral activity against dengue virus. *Sci. Rep.* 4:5452. 10.1038/srep05452 PMC4071309 24965553

[B35] Nazarian R. Shi H. Wang Q. Kong X. Koya R. C. Lee H. (2010). Melanomas acquire resistance to B-RAF(V600E) inhibition by RTK or N-RAS upregulation. *Nature* 468 973–977. 10.1038/nature09626 21107323 PMC3143360

[B36] Omholt K. Platz A. Kanter L. Ringborg U. Hansson J. (2003). NRAS and BRAF mutations arise early during melanoma pathogenesis and are preserved throughout tumor progression. *Clin. Cancer Res.* 9 6483–6488. 14695152

[B37] Poulikakos P. I. Zhang C. Bollag G. Shokat K. M. Rosen N. (2010). RAF inhibitors transactivate RAF dimers and ERK signalling in cells with wild-type BRAF. *Nature* 464 427–430. 10.1038/nature08902 20179705 PMC3178447

[B38] Pusapati R. V. Daemen A. Wilson C. Sandoval W. Gao M. Haley B. (2016). mTORC1-dependent metabolic reprogramming underlies escape from glycolysis addiction in cancer cells. *Cancer Cell* 29 548–562. 10.1016/j.ccell.2016.02.018 27052953

[B39] Salmond R. J. (2018). mTOR regulation of glycolytic metabolism in T cells. *Front. Cell Dev. Biol.* 6:122. 10.3389/fcell.2018.00122 30320109 PMC6167959

[B40] Stover J. F. Belli A. Boret H. Bulters D. Sahuquillo J. Schmutzhard E. (2014). Nitric oxide synthase inhibition with the antipterin VAS203 improves outcome in moderate and severe traumatic brain injury: a placebo-controlled randomized Phase IIa trial (NOSTRA). *J. Neurotrauma* 31 1599–1606. 10.1089/neu.2014.3344 24831445

[B41] Sun Q. Chen X. Ma J. Peng H. Wang F. Zha X. (2011). Mammalian target of rapamycin up-regulation of pyruvate kinase isoenzyme type M2 is critical for aerobic glycolysis and tumor growth. *Proc. Natl. Acad. Sci. U.S.A.* 108 4129–4134. 10.1073/pnas.1014769108 21325052 PMC3054028

[B42] Takahashi H. Chen M. C. Pham H. Angst E. King J. C. Park J. (2011). Baicalein, a component of *Scutellaria baicalensis*, induces apoptosis by Mcl-1 down-regulation in human pancreatic cancer cells. *Biochim. Biophys. Acta* 1813 1465–1474. 21596068 10.1016/j.bbamcr.2011.05.003 PMC3123440

[B43] Thomas N. E. Edmiston S. N. Alexander A. Groben P. A. Parrish E. Kricker A. (2015). Association between NRAS and BRAF mutational status and melanoma-specific survival among patients with higher-risk primary melanoma. *JAMA Oncol.* 1 359–368. 26146664 10.1001/jamaoncol.2015.0493 PMC4486299

[B44] Thumar J. Shahbazian D. Aziz S. A. Jilaveanu L. B. Kluger H. M. (2014). MEK targeting in N-RAS mutated metastatic melanoma. *Mol. Cancer* 13:45. 10.1186/1476-4598-13-45 24588908 PMC3945937

[B45] Ugurel S. Thirumaran R. K. Bloethner S. Gast A. Sucker A. Mueller-Berghaus J. (2007). B-RAF and N-RAS mutations are preserved during short time in vitro propagation and differentially impact prognosis. *PLoS One* 2:e236. 10.1371/journal.pone.0000236 17311103 PMC1794595

[B46] Villanueva J. Vultur A. Lee J. T. Somasundaram R. Fukunaga-Kalabis M. Cipolla A. K. (2010). Acquired resistance to BRAF inhibitors mediated by a RAF kinase switch in melanoma can be overcome by cotargeting MEK and IGF-1R/PI3K. *Cancer Cell* 18 683–695. 10.1016/j.ccr.2010.11.023 21156289 PMC3026446

[B47] Wang C. Z. Zhang C. F. Chen L. Anderson S. Lu F. Yuan C. S. (2015). Colon cancer chemopreventive effects of baicalein, an active enteric microbiome metabolite from baicalin. *Int. J. Oncol.* 47 1749–1758. 10.3892/ijo.2015.3173 26398706 PMC4599184

[B48] Wang Z. Ma L. Su M. Zhou Y. Mao K. Li C. (2018). Baicalin induces cellular senescence in human colon cancer cells via upregulation of DEPP and the activation of Ras/Raf/MEK/ERK signaling. *Cell Death Dis.* 9:217. 10.1038/s41419-017-0223-0 PMC5833439 29440765

[B49] Warburg O. (1956). On the origin of cancer cells. *Science* 123 309–314. 13298683 10.1126/science.123.3191.309

[B50] Ward P. S. Thompson C. B. (2012). Metabolic reprogramming: a cancer hallmark even warburg did not anticipate. *Cancer Cell* 21 297–308. 10.1016/j.ccr.2012.02.014 22439925 PMC3311998

[B51] Xiao J. R. Do C. W. To C. H. (2014). Potential therapeutic effects of baicalein, baicalin, and wogonin in ocular disorders. *J. Ocul. Pharmacol. Ther.* 30 605–614. 10.1089/jop.2014.0074 25280175

[B52] Yang H. Jiang X. Li B. Yang H. J. Miller M. Yang A. (2017). Mechanisms of mTORC1 activation by RHEB and inhibition by PRAS40. *Nature* 552 368–373. 10.1038/nature25023 29236692 PMC5750076

[B53] Ye J. Huang X. Hsueh E. C. Zhang Q. Ma C. Zhang Y. (2012). Human regulatory T cells induce T-lymphocyte senescence. *Blood* 120 2021–2031. 10.1182/blood-2012-03-416040 22723548 PMC3437594

[B54] Ye J. Ma C. Hsueh E. C. Dou J. Mo W. Liu S. (2014). TLR8 signaling enhances tumor immunity by preventing tumor-induced T-cell senescence. *EMBO Mol. Med.* 6 1294–1311. 10.15252/emmm.201403918 25231413 PMC4287933

[B55] Yoshida G. J. (2015). Metabolic reprogramming: the emerging concept and associated therapeutic strategies. *J. Exp. Clin. Cancer. Res.* 34:111. 10.1186/s13046-015-0221-y PMC4595070 26445347

[B56] Yu C. Zhang Z. Zhang H. Zhen Z. Calway T. Wang Y. (2013). Pretreatment of baicalin and wogonoside with glycoside hydrolase: a promising approach to enhance anticancer potential. *Oncol. Rep.* 30 2411–2418. 10.3892/or.2013.2726 24026776 PMC3820585

[B57] Zhang H. B. Lu P. Guo Q. Y. Zhang Z. H. Meng X. Y. (2013). Baicalein induces apoptosis in esophageal squamous cell carcinoma cells through modulation of the PI3K/Akt pathway. *Oncol. Lett.* 5 722–728. 10.3892/ol.2012.1069 23420294 PMC3572959

[B58] Zhdanov A. V. Okkelman I. A. Collins F. W. Melgar S. Papkovsky D. B. (2015). A novel effect of DMOG on cell metabolism: direct inhibition of mitochondrial function precedes HIF target gene expression. *Biochim. Biophys. Acta* 1847 1254–1266. 10.1016/j.bbabio.2015.06.016 26143176

