# Corrigendum: Baicalein and Baicalin Promote Melanoma Apoptosis and Senescence *via* Metabolic Inhibition

**DOI:** 10.3389/fcell.2022.876000

**Published:** 2022-03-28

**Authors:** Lan Huang, Bo Peng, Yash Nayak, Cindy Wang, Fusheng Si, Xia Liu, Jie Dou, Huaxi Xu, Guangyong Peng

**Affiliations:** ^1^ Department of Immunology, School of Medicine, Jiangsu University, Zhenjiang, China; ^2^ Division of Infectious Diseases, Allergy and Immunology, Department of Internal Medicine, School of Medicine, Saint Louis University, Saint Louis, MO, United States; ^3^ State Key Laboratory of Natural Medicines, School of Life Science and Technology, China Pharmaceutical University, Nanjing, China

**Keywords:** baicalein, baicalin, melanoma, N-RAS, B-RAF, apoptosis, senescence, glucose metabolism

In the original article, there were mistakes in Figures 2B,C as published. “We misused some data for both cell lines in Figure 2B. In addition, we made mistakes and uploaded the wrong images for some time points and treatments in Figure 2C.” The corrected Figure 2 appears below.

**FIGURE 2:**
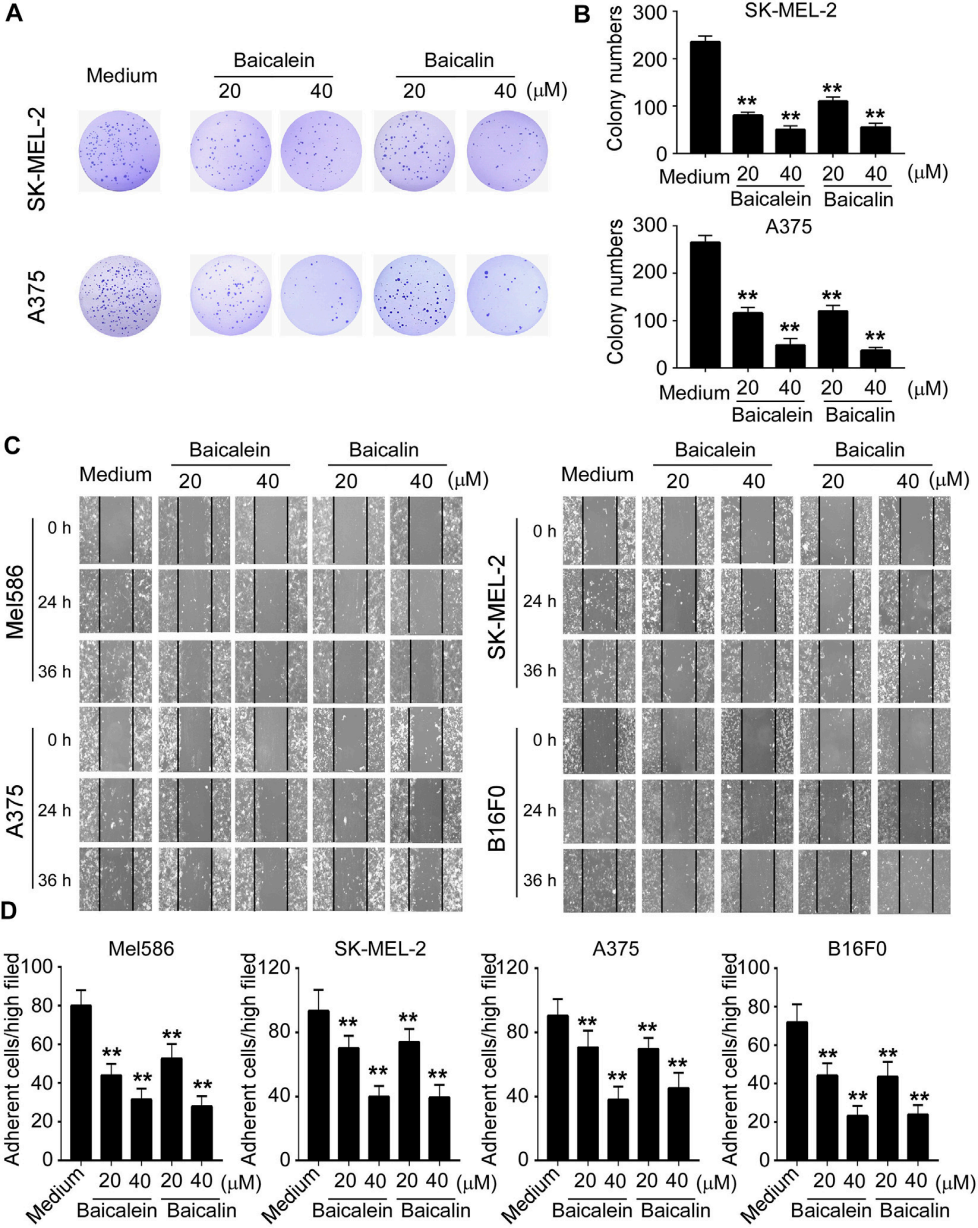
Baicalein and baicalin inhibit melanoma cell colony formation, migration and adhesion. **(A,B)** Baicalein and baicalin treatments dramatically decreased the numbers and sizes of tumor colonies in SK-MEL-2 and A375 cells. 200–500 per well of melanoma cells pre-treated with the indicated concentrations of baicalein or baicalin, were seeded in 6-well plates for culture, and cell colonies counted after 10–14 days of culture. Results shown in the histogram **(B)** are summaries of mean ± SD from three independent experiments. **p < 0.01 compared with the medium control group. **(C)** Different concentrations of baicalein and baicalin treatments in both human and mouse melanoma cells significantly inhibited tumor cell migration compared with the medium control group at 24 and 36 h time points in the wound closure assays. Data shown are representatives from three independent experiments with similar results. **(D)** Baicalein and baicalin treatments suppressed the adhesion of melanoma cells. Both human and mouse melanoma cells pretreated with the indicated concentrations of baicalein and baicalin were cultured in the fibronectin-coated plates for 45 min. Adherent cells were counted and averaged in 10 fields at high (×400) magnification with a microscope. Results shown are summaries of mean ± SD from three independent experiments with similar results **p < 0.01 compared with the medium control group.

The authors apologize for the errors and state that this does not change the scientific conclusions of the article in any way. The original article has been updated.

