# Supplementary figures and images for: Baicalein and Baicalin Promote Melanoma Apoptosis and Senescence via Metabolic Inhibition
Source: Front Cell Dev Biol. 2020 Aug 25;8:836. doi: 10.3389/fcell.2020.00836 (PMC7477299; doi:10.3389/fcell.2020.00836)

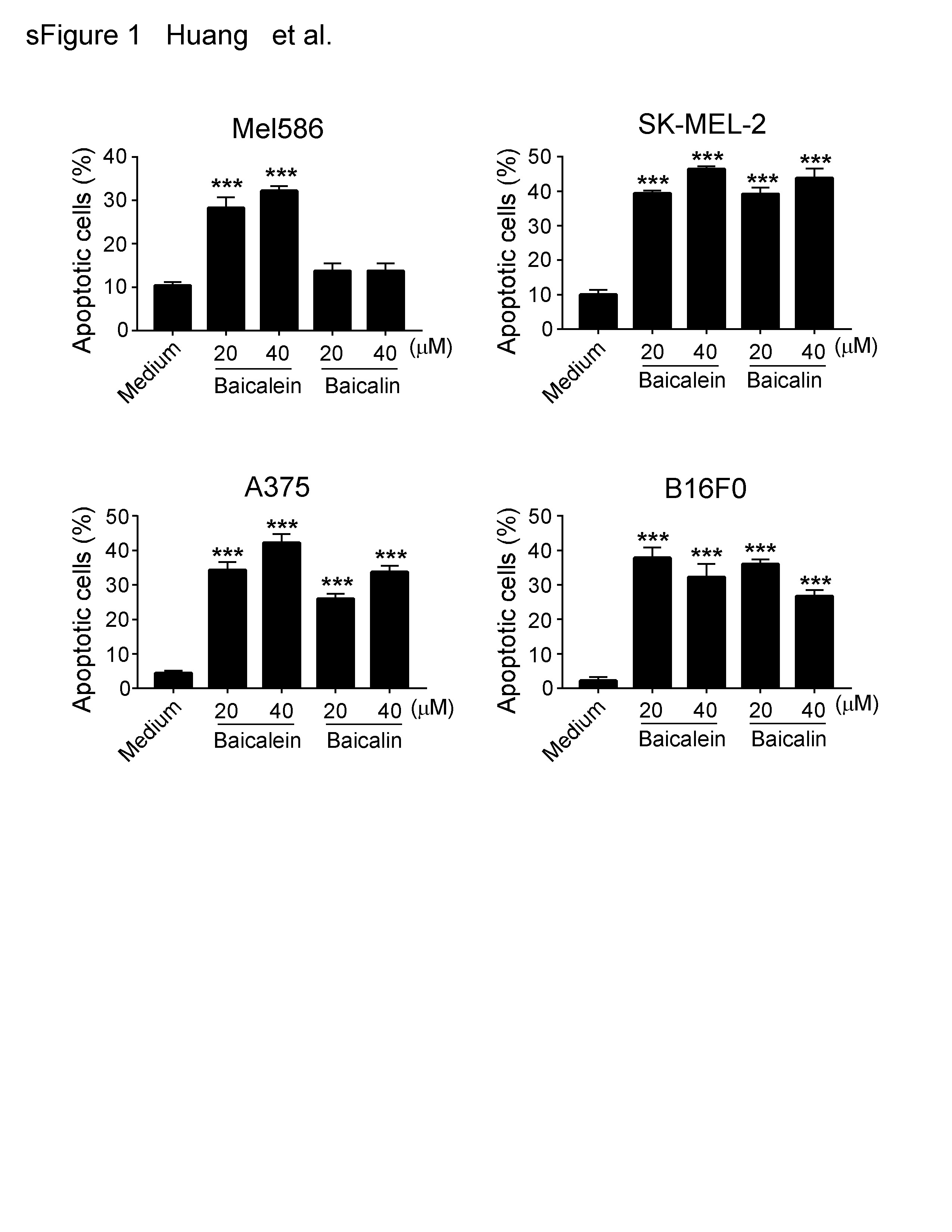

Supplement: FIGURE S1 — Increased apoptotic cell populations are induced in both human and mouse melanoma cells after treatments with baicalein and baicalin. Tumor cells were cultured in the presence of the indicated concentrations of baicalein and baicalin for 36 h. Apoptosis in treated tumor cells was analyzed after staining with PE-labeled Annexin V and 7-AAD. Results shown in the histogram are summaries of mean ± SD from three independent experiments. ∗∗∗p < 0.001, compared with the medium control group. [file Image_1.JPEG]

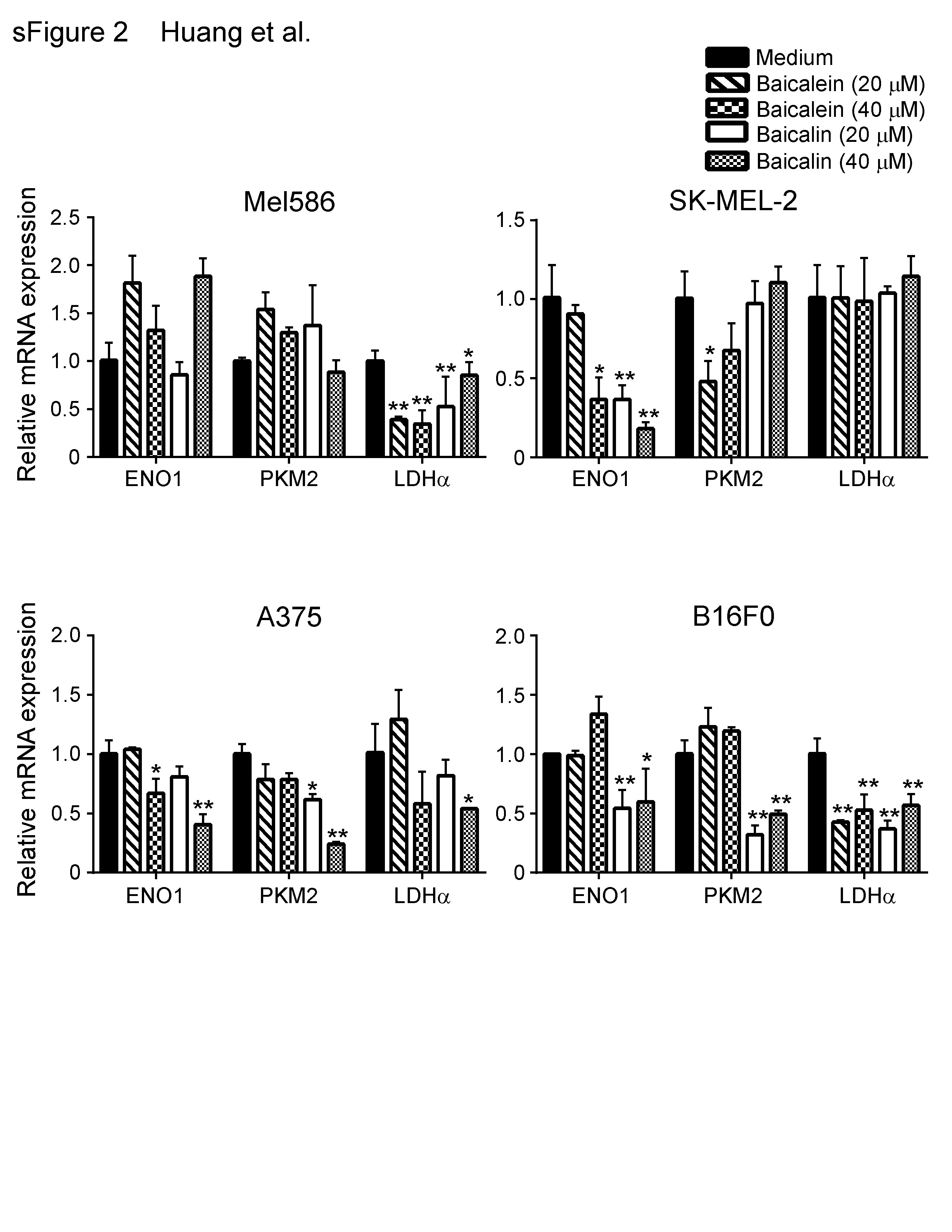

Supplement: FIGURE S2 — Baicalein and baicalin treatments significantly down-regulate gene expression levels of key glycolytic enzymes in melanoma cells. Both human and mouse Melanoma cells were treated with or without the indicated concentrations of baicalein and baicalin for 72 h. Total RNA was isolated from the tumor cells and analyzed by Real-time PCR. The expression levels of each gene were normalized to β-actin expression levels and adjusted to the levels in untreated tumor cells (medium). Data shown in different melanoma cells are mean ± SD from three independent experiments. ∗p < 0.05 and ∗∗p < 0.01, compared with the medium only group. [file Image_2.JPEG]

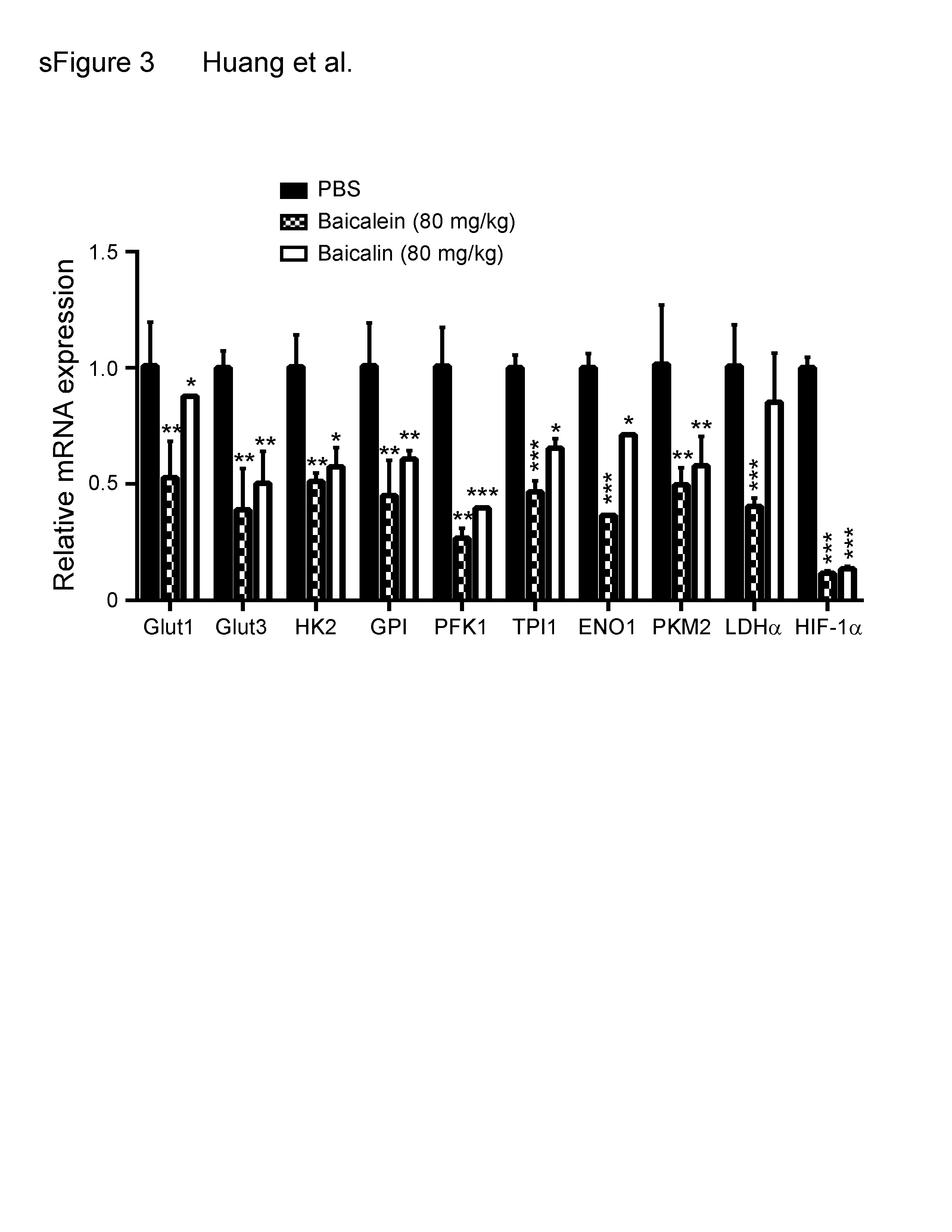

Supplement: FIGURE S3 — Baicalein and baicalin treatments down-regulate gene expression levels of key glycolytic enzymes in B16F0 tumor cells in vivo. B16F0 cells (1 × 10^5^/mouse) were subcutaneously injected into NSG mice. After 4 days post tumor injection (tumor size reached around 5 × 5 mm), the tumor-bearing mice were administrated with baicalein (80 mg/kg), baicalin (80 mg/kg), or PBS control through intraperitoneal injection, respectively at every other day for 2 weeks. At the end of experiments, tumor issues were grinded and total RNA was extracted. The expression levels of each gene were normalized to β-actin expression levels and adjusted to the levels in PBS treated group (set as 1). Results shown are mean ± SD from four mice per group. ∗p < 0.05, ∗∗p < 0.01, and ∗∗∗p < 0.001, compared with the PBS treatment control group using unpaired t-test. [file Image_3.JPEG]
