# Supplementary material for: Baicalein and Baicalin Promote Melanoma Apoptosis and Senescence via Metabolic Inhibition
Source: Front Cell Dev Biol. 2020 Aug 25;8:836. doi: 10.3389/fcell.2020.00836 (PMC7477299; doi:10.3389/fcell.2020.00836)
Supplement: Supplementary file 4 [file Table_1.pdf]

**Supplemental Table 1. Primers used for Real-time quantitative RT-PCR**

| Genes | Primers |
|---|---|
| Hu-Glut1 Forward | ATTGGCTCCGGTATCGTCAAC |
| Hu-Glut1 Reverse | GCTCAGATAGGACATCCAGGGTA |
| Hu-Glut3 Forward | GCTCTCTGGGATCAATGCTGTGT |
| Hu-Glut3 Reverse | CTTCCTGCCCTTTCCACCAGA |
| Hu-HK2 Forward | AACAGCCTGGACGAGAGCAT |
| Hu-HK2 Reverse | GCCAACAATGAGGCCAACTT |
| Hu-GPI Forward | GATGGTAGCTCTCTGCAGCC |
| Hu-GPI Reverse | GCCATGGCGGGACTCTTG |
| Hu-PFK Forward | GGCAGCCATGCATAAAGACG |
| Hu-PFK Reverse | AAGCTTCCCCAGCTGTTCTC |
| Hu-TPI Forward | AGGCATGTCTTTGGGGAGTC |
| Hu-TPI Reverse | AGTCCTTCACGTTATCTGCGA |
| Hu-ENO1 Forward | CGCCTTAGCTAGGCAGGAAG |
| Hu-ENO1 Reverse | GGTGAACTTCTAGCCACTGGG |
| Hu-PKM2 Forward | ACGAGAACATCCTGTGGCTG |
| Hu-PKM2 Reverse | AGGAAGTCGGCACCTTTCTG |
| Hu-LDHα Forward | AGCTGTTCCACTTAAGGCCC |
| Hu-LDHα Reverse | TGGAACCAAAAGGAATCGGGA |
| Hu-HIF-1a Forward | GAACGTCGAAAAGAAAAGTCTCG |
| Hu-HIF-1a Reverse | CCTTATCAAGATGCGAACTCACA |
| Hu-β-actin Forward | TGGCACCCAGCACAATGAA |
| Hu-β-actin Reverse | CTAAGTCATAGTCCGCCTAGAAGCA |
| Mu-Glut1 Forward | CAGTTCGGCTATAACACTGGTG |
| Mu-Glut1 Reverse | GCCCCCGACAGAGAAGATG |
| Mu-Glut3 Forward | CTTTGGCAGACGCAACTCTAT |
| Mu-Glut3 Reverse | ACCAGAATCCCAACAACGATG |
| Mu-HK2 Forward | TGATCGCCTGCTTATTCACGG |
| Mu-HK2 Reverse | AACCGCCTAGAAATCTCCAGA |
| Mu-GPI Forward | TCCCCTGAGACTTCCCTCTTT |
| Mu-GPI Reverse | CGAGAAACCACTCCTTTGCTG |
| Mu-PFK Forward | TGTGGTCCGAGTTGGTATCTT |
| Mu-PFK Reverse | GCACTTCCAATCACTGTGCC |
| Mu-TPI Forward | CCAGGAAGTTCTTCGTTGGGG |
| Mu-TPI Reverse | CAAAGTCGATGTAAGCGGTGG |
| Mu-ENO1 Forward | AGAGTGGGAGGCGCTTAGT |
| Mu-ENO1 Reverse | ATGGCGAATTTCTGGCAGTAG |
| Mu-PKM2 Forward | GTGGCTCGGCTGAATTTCTCT |
| Mu-PKM2 Reverse | CACCGCAACAGGACGGTAG |
| Mu-LDH Forward | TGTCTCCAGCAAAGACTACTGT |
| Mu-LDH Reverse | GACTGTACTTGACAATGTTGGGA |
| Mu-HIF-1a Forward | GATGACGGCGACATGGTTTAC |
| Mu-HIF-1a Reverse | CTCACTGGGCCATTTCTGTGT |
| Mu-β-actin Forward | GATCAAGATCATTGCTCCTCCTG |
| Mu-β-actin Reverse | AGGGTGTAAAACGCAGCTCA |
